# Probing fibronectin adsorption on chemically defined surfaces by means of single molecule force microscopy

**DOI:** 10.1038/s41598-020-72617-z

**Published:** 2020-09-24

**Authors:** Evangelos Liamas, Richard A. Black, Paul A. Mulheran, Robert Tampé, Ralph Wieneke, Owen R. T. Thomas, Zhenyu J. Zhang

**Affiliations:** 1 School Chemical Engineering, University of Birmingham, Edgbaston, Birmingham, B15 2TT UK; 2 Department of Biomedical Engineering, University of Strathclyde, 106 Rottenrow, Glasgow, G4 0NW UK; 3 Department of Chemical and Process Engineering, University of Strathclyde, 75 Montrose Street, Glasgow, G1 1XJ UK; 4 Institute of Biochemistry, Biocenter, Goethe University Frankfurt, Max von Laue Strasse 9, 60438 Frankfurt/Main, Germany; 5 Present Address: School of Food Science and Nutrition, University of Leeds, Woodhouse Lane, Leeds, LS2 9JT UK

**Keywords:** Molecular biophysics, Nanoscale biophysics, Single-molecule biophysics, Macromolecules and clusters, Biomedical engineering, Biosurfaces, Scanning probe microscopy

## Abstract

Atomic force microscope (AFM) based single molecule force spectroscopy (SMFS) and a quartz crystal microbalance (QCM) were respectively employed to probe interfacial characteristics of fibronectin fragment FNIII^8–14^ and full-length fibronectin (FN) on CH_3_–, OH–, COOH–, and NH_2_-terminated alkane-thiol self-assembled monolayers (SAMs). Force-distance curves acquired between hexahistidine-tagged FNIII^8–14^ immobilised on *tris*NTA-Ni^2+^ functionalized AFM cantilevers and the OH and COOH SAM surfaces were predominantly ‘loop-like’ (76% and 94% respectively), suggesting domain unfolding and preference for ‘end-on’ oriented binding, while those generated with NH_2_ and CH_3_ SAMs were largely ‘mixed type’ (81% and 86%, respectively) commensurate with unravelling and desorption, and ‘side-on’ binding. Time-dependent binding of FN to SAM-coated QCM crystals occurred in at least two phases: initial rapid coverage over the first 5 min; and variably diminishing adsorption thereafter (5–70 min). Loading profiles and the final hydrated surface concentrations reached (~ 950, ~ 1200, ~ 1400, ~ 1500 ng cm^−2^ for CH_3_, OH, COOH and NH_2_ SAMs) were consistent with: space-filling ‘side-on’ orientation and unfolding on CH_3_ SAM; greater numbers of FN molecules arranged ‘end-on’ on OH and especially COOH SAMs; and initial ‘side-on’ contact, followed by either (1) gradual tilting to a space-saving ‘end-on’ configuration, or (2) bi-/multi-layer adsorption on NH_2_ SAM.

## Introduction

Key to the successful integration of all medical ‘biomaterials’ in human body, in contact with tissue and blood, be they synthetic or natural, is their ‘biocompatibility’ defined by Ratner^1^ as the ‘ability of biomaterials to: (1) locally trigger and guide wound healing and tissue regeneration; and (2) reside in the body for long periods of time with only low degrees of inflammatory reaction’. Their contact with proteins is particularly important in determining the response of the surrounding tissues, given that they in turn mediate the attachment of cells^2^. The conformation adopted by surface adsorbed proteins has been identified as a primary determinant of cell binding; while certain conformations promote cell binding and successful integration of the biomaterial, others inhibit it, inducing an immune response, which ultimately leads to implant rejection^3,4^. The form that given proteins assume on interacting with biomaterials is strongly influenced by the surface chemistry of the latter^5–7^. It is for this reason that medical devices are commonly modified with a coating that is chemically and biologically compatible with the intended target tissue. For example, coating a medical grade material with hydroxyapatite enhances its osseointegration, i.e. accelerates and increases bone contact with the implant surface^8^. Continued advance in surface coatings for biomaterials is required however; in the case of bone and dental implants to improve osseous integration by booting osteoblast adhesion^9–11^. The development of medical grade materials with improved biocompatibility requires comprehensive understanding of the effects of surface chemistry on protein adsorption. Less obvious, though nevertheless important, is the need to quantify molecular interactions between differently functionalized surfaces and adsorbing proteins. With the growing availability of powerful non-destructive quantitative methods such as atomic force microscope based single-molecule force spectroscopy (SMFS)^12,13^, and high resolution mass sensing on a Quartz Crystal Microbalance, QCM^14,15^, it is now possible to analyze/measure interactions between proteins and biomaterials at the molecular level.

One of the main proteins mediating the interaction of cells with surfaces is fibronectin (FN), a major component of the extracellular matrix in many tissues^16,17^. FN is a complex high molecular weight (470–500 kDa) glycoprotein dimer that binds other extracellular matrix proteins (e.g. collagen, fibrin, syndecans, tenacins), and importantly, integrin adhesion molecules, a large family of cell membrane spanning receptor proteins^16,18,19^. The nearly identical subunits are linked by a pair of C-terminal disulfide bonds, each comprising multiple homologous modules (termed FNI, FNII and FNIII) arranged into many distinct functional and binding domains^20,21^. FNIII domains 9 (FNIII^9^) and 10 (FNIII^10^) are responsible for cell binding. The crucial attachment site for cell surface anchored FN receptors (e.g*.* integrins α_5_β_1_ and α_V_β_3_) is the RGD (Arg-Gly-Asp) sequence^22,23^ in FN^10^, while the synergy site PHSRN (Pro-His-Ser-Arg-Asn)^24–26^ located in FNIII^9^, modulates the association of FN with integrins. SMFS has been applied in the study of FN’s mechanical stability and interactions with other molecules, notably integrins. Collectively, these investigations reveal FNIII modules containing the cell-binding site as weakest in the mechanical sense^27–30^ and that force-induced dissociation of the complex formed between α_5_β_1_ integrin and fibronectin fragment FNIII^7–10^ requires overcoming two activation barriers, indicating that integrin activation involves cooperative interaction of the FNIII^10^ RGD and FNIII^9^ PHSRN synergy sites^31^.

SMFS has also been used, albeit rarely to date, to interrogate the interaction of FN with homogeneous chemically-defined surfaces. In a notable example, Meadows and Walker^32^ employed SMFS to investigate the adsorption of full-length FN supplied at two different concentrations on a wide range of defined surfaces (including mica, gold, polyethylene glycol and SAMs terminated with methyl, hydroxyl or carboxyl functions). The authors reported that at high bulk phase FN concentration, i.e. high FN challenge to the surface (aggregate state), adsorbed FN adopted a looser conformation and exhibited weaker binding on hydrophilic *cf.* hydrophobic surfaces, whereas at low solution phase concentration (low FN challenge to the surface, semi-aggregate state) the larger number of substrate contacts available to each adsorbed FN molecule resulted in their adopting more rigid conformations *cf.* the corresponding aggregate state. However, because FN was pre-adsorbed onto the target surfaces rather than anchored onto AFM cantilevers the observed interactions were non-specific in nature, with only 15–20% of the acquired data displaying pulling events. In earlier work from the same laboratory, Meadows et al.^33^ investigated desorption of full-length FN from a negatively charged mica surface as a function of prior FN loading; here too FN was pre-adsorbed on the test surface not attached to the AFM tip. At high FN challenge to the surface force-distance curves exhibited successive rupture events indicative of protein unfolding, while at low FN challenge FN partially denatured upon adsorption, generating force-distance curves lacking dominant pulling events.

QCM has also been used to study the adsorption of proteins including fibronectin on chemically defined surfaces. Dickerson et al.^34^ employed QCM to investigate the two-stage adsorption under physiological conditions of a recombinant FN-binding outer membrane protein fragment, rTp0483, on four differently terminated SAMs, followed by FN. They reported that initial binding of rTp0483 was greater on the negatively charged COOH SAM *cf.* all others tested, i.e. NH_2_, OH and CH_3_ SAMs (a finding consistent with the fragment’s net positive charge under experimental conditions); and further that rTp0483 adsorption was also more uniform on the negatively charged SAM *cf.* other surfaces (revealed by AFM). These two factors combined explained the much higher FN loadings on ‘rTp0483 primed’ COOH SAM *cf.* other fragment-primed SAMs. In a different study, using COOH and NH_2_ SAMs blended in different ratios to control the surface chemistry of gold-coated QCM crystals, Lin et al.^35^ reported that despite carrying a small net negative charge (− 5.7 mV) under experimental binding conditions (phosphate buffered saline) FN adsorbed on both negatively and positively charged surfaces. Interestingly, the authors’ observed greater adsorption on strongly negatively charged SAMs *cf.* moderately negative and weakly positive SAMs, which they attributed to a fine balance of surface charge induced polarization of the protein, short-range holding forces and simple electrostatic interactions.

In this study we have employed SMFS and QCM to respectively probe the interactions of fibronectin domain FNIII^8–14^ and the full-length FN molecule with the same four chemically defined surfaces (i.e. NH_2_-, COOH-, OH- and CH_3_-terminated SAMs) to derive information on unfolding and denaturation, preferential binding orientation in the adsorbed state, surface loading, and binding kinetics. Specifically, we have used SMFS to extract information on the forces developed between FNIII^8–14^ immobilized in various forms on gold AFM tips and the different SAMs, QCM to extract complementary information (kinetics, loading, changes in binding orientation as function of time/loading) on the binding of free FN to gold-coated QCM crystals modified with the same SAMs. Note, a full-length human FN was used in QCM studies in place of FNIII^8–14^; given Michael et al.^36^ finding of very similar adsorption of fibronectin domain FNIII^7–10^ and the full-length molecule and because the costs of using FNIII^8–14^ in QCM were prohibitive.

## Materials and methods

### Materials

Full-length human plasma fibronectin (fc010) and recombinant human fibronectin fragment 3 protein, CF (FN-050; Carrier free histidine-tagged FNIII^8–14^ fragment) were obtained from Millipore Limited (Hertfordshire, UK) and R&D systems (Minneapolis, Minnesota, USA) respectively, and the *tris*NTA-EG3-C16-SH linker was synthesized as described previously^37–39^. Gold-coated QCM crystals were supplied by openQCM (Novaetech S.r.l., Napoli, Italy) and gold-coated AFM chips (NPG-10) were purchased from Bruker (UK) Limited (Coventry, UK). The chemicals/reagents, ethanol (> 99.5% for HPLC), hydrogen peroxide solution (30% w/w in water), concentrated sulfuric acid (99.999%), 1-hexanethiol (HS–(CH_2_)_5_–CH_3_)_,_ 6-mercaptohexanoic acid (HS–(CH_2_)_5_–COOH), 6-amino-1-hexanethiol (HS–(CH_2_)_6_–NH_2_), 6-mercapto-1-hexanol (HS–(CH_2_)_6_–OH), 11-mercapto-1-undecanol (HS–(CH_2_)_11_–OH), nickel (II) chloride and ethylenediaminetetraacetic acid (EDTA) were obtained from Sigma-Aldrich (Dorset, UK), whereas tris(2-carboxyethyl)phosphine (TCEP), 4-(2-hydroxyethyl)-1-piperazineethanesulfonic acid (HEPES), phosphate buffered saline (PBS) tablets and sodium chloride were acquired from Fisher Scientific UK Ltd. (Loughborough, UK). All solutions were prepared using deionized water of HPLC grade (Sigma Aldrich, Dorset, UK).

### Preparation of self-assembled monolayers (SAMs)

Prior to their use as substrates for SAMs gold-coated QCM crystals were cleaned with freshly prepared piranha solution (7:3 mix of concentrated sulfuric acid and 30% w/w hydrogen peroxide). Hydroxyl, methyl, amine and carboxyl terminated SAMs were prepared according to Sigma Aldrich Technical Bulletin AL-266. Briefly, this involved immersing gold-coated QCM sensors in ethanolic solutions of different 2 mM alkanethiol (HS–(CH_2_)_5_–CH_3_, HS–(CH_2_)_5_–COOH, HS–(CH_2_)_6_–NH_2_, HS–(CH_2_)_6_–OH) solutions for 24 h. Self-assembly was terminated by rinsing the modified QCM sensors for 120 s in ethanol in a sonicating bath, before drying under nitrogen. Static contact angle measurements of the resulting SAMs, hereafter identified by the terminal function they present (CH_3_, COOH, NH_2_ and OH SAMs) were made using a contact angle goniometer (Biolin Scientific, Manchester, UK). Values of 101 ± 1°, 50 ± 1°, 35 ± 1° and 11 ± 1° (n = 3, mean ± SD) found for the CH_3_, NH_2_, OH and COOH, terminated SAMs respectively are in agreement with previous reports^40–43^.

### Zeta potential measurement

A Zetasizer Nano ZS (Malvern Panalytical Ltd, Malvern, UK) was used to determine the zeta potential, ζ, of FNIII^8–14^ in 1 M HEPES buffer at pH 7.4, and value of − 14 ± 1 mV was found.

### Functionalization of AFM cantilevers

FNIII^8–14^ fragment derivatized AFM cantilevers were prepared in stepwise manner using a variation of Gruber’s protocol^44^, exploiting sulfur–gold bonding and immobilized metal chelate–histidine binding interactions^37,38,45^. Briefly this involved: (1) cleaning gold-coated AFM chips with UV/ozone; (2) immersing the cleaned AFM chips in an ethanolic solution containing 2 mM of 11-mercapto-1-undecanol, 0.02 mM of *tris*NTA-EG_3_-C_16_-SH linker, 2 mM EDTA and 2 mM TCEP for 24 h; (3) successive rinsing of the ‘*tris*NTA-EG_3_-C_16_/undecanol SAM’ modified AFM chips with absolute ethanol followed by 40 mM HEPES pH 7.4 buffer supplemented with 40 mM EDTA and 85 mM NaCl, before drying under nitrogen; (4) incubating in 200 μL buffer containing 0.5 µM FNIII^8–14^, 200 µM nickel (II) chloride at 37 °C for 1.5 h; and finally, rinsing the FNIII^8–14^ with 1 M HEPES solution in readiness for AFM experiments.

### Force spectroscopy

Force measurements were performed in 1 M HEPES buffer pH 7.4 at room temperature in the fluid cell of a Nanowizard II atomic force microscope (JPK Instruments AG, Berlin, Germany). The AFM was loaded with fully functionalized cantilevers (featuring chemically attached linker and FNIII^8–14^ fragment designated ‘gold-linker-FNIII^8–14^′) and also with the following control cantilevers: gold coated AFM cantilever (‘gold’); FNIII^8–14^ physisorbed on bare cantilever (‘gold-FNIII^8–14^’); and cantilevers functionalized with *tris*NTA-EG_3_-C_16_-SH linker (‘gold-linker’).

Prior to experiments, AFM cantilevers were calibrated as detailed by Hutter and Bechhoefer^46^. A force of magnitude 1 nN was applied to the tip as it approached the SAM surface with a velocity of 1 µm s^−1^. Once the tip had reached the surface, contact was maintained for 3 s before it was retracted and the process repeated. The force-distance (FD) curves were acquired from 16 different positions (4 × 4 grid) on the surface used, while the distance between each position was 10 μm. The tests were repeated four times with different AFM cantilevers and surfaces to ensure the reproducibility of the measurements.

### Surface adsorption

A quartz crystal microbalance (OpenQCM, Novaetech S.r.l, Italy) was employed to determine the adsorption of full-length human plasma fibronectin on the different SAMs surfaces. SAM-coated QCM crystals were loaded onto the QCM before introducing the fibronectin solution (25 µg mL^−1^ in PBS pH 7.4) at a flow of 1 µL s^−1^ using an Ismatec IPC-N 4 peristaltic pump (Ismatec, Wertheim, Germany). Masses of adsorbed hydrated protein were calculated using the Sauerbrey equation^47^ (Eq. 1):

1 $$\Delta f_{m} = - \frac{{2f_{0}^{2} }}{{A\sqrt {\rho_{q} \mu_{q} } }} \cdot \Delta m = - C_{f} \cdot \Delta m$$

here Δ*f*_*m*_ is the measured resonant frequency (Hz), *f*_*0*_ is the intrinsic resonant frequency of the unloaded crystal (Hz), Δ*m* is the mass change (g), *A* is the mass sensitive area of the electrode (cm^2^), *ρ*_*q*_ is the density of quartz (2.648 g cm^3^), *µ*_*q*_ is the shear modulus of the quartz crystal (2.947 × 10^11^ g cm^−1^ s^−2)^ and *C*_*f*_ is the quartz sensitivity factor or constant (Hz g^−1^). The negative sign in the formula implies that an increase in mass on the surface of the crystal results in a reduction of the measured resonant frequency. A total of seven measurements were performed for each SAM surface.

### Data handling

All data is presented as mean values ± the standard error of the mean. An analysis of variance (ANOVA) was used to evaluate the significance of the measured parameters.

## Results and discussion

### Force spectroscopy measurements

The functionalization of AFM tips with the FNIII^8–14^ fragment is a two-step process, involving: (1) initial attachment of a heterobifunctional linker (*tris*NTA-EG_3_-C_16_-SH) via its thiol end to the gold-coated AFM tip; followed by (2) anchoring of the FNIII^8–14^ to the Ni(II)-loaded chelate moiety at the end of the linker via the fragment’s His-tag. To identify the contribution from each chemical species, force measurements were performed between the AFM tip and the SAMs surfaces at every step of the functionalization. Prior to examining FD curves of the chemically attached FNIII^8–14^ (‘gold-linker-FNIII^8–14^′) with the different SAM modified surfaces a hierarchical series of tests were conducted using bare (‘gold’), physisorbed FNIII^8–14^ (‘gold-FNIII^8–14^’), and *tris*NTA-EG_3_-C_16_-SH linker (‘gold-linker’) AFM cantilevers.

### Interaction between ‘gold’ AFM tips and SAMs

**Figure 1:**
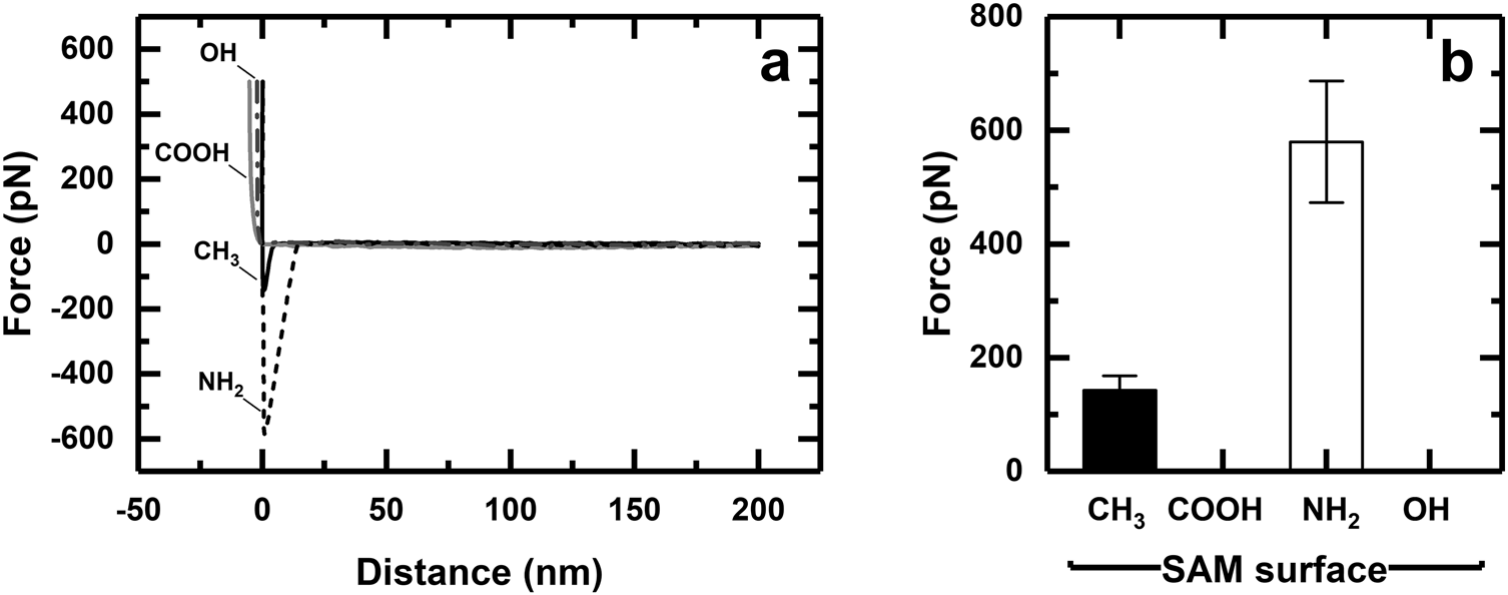
Plots of (**a**) characteristic force vs. distance (FD) retraction curves and (**b**) mean adhesion force for bare gold AFM tips against four different SAMs in 1 M HEPES buffer pH 7.5. The data in (**b**) represent the mean values ± standard error of n = 64 force curves.

**Figure 2:**
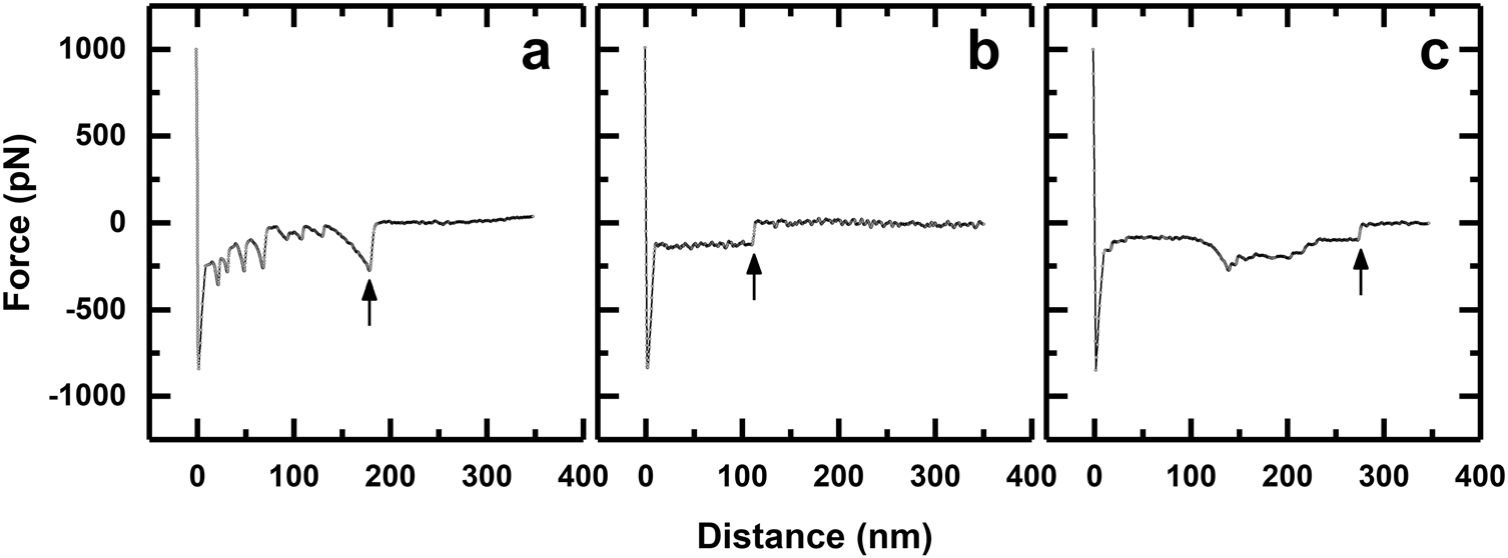
Characteristic FD curves illustrating examples of: (**a**) a ‘loop-like’ retraction event indicating protein unfolding; (**b**) a ‘train-like’ retraction event implying protein desorption; and (**c**) a ‘mixed’ retraction event inferring desorption and unfolding. Desorption force is determined from the last peak in the retraction curve at which point (↑), the ‘desorption distance’, the protein detaches from the surface.

Figure 1a,b respectively show representative FD retraction curves of bare gold-coated AFM chips contacting the four different SAM surfaces, and the mean forces that are required to separate them. Adhesion was noted to two of the four SAMs, i.e. methyl and amine terminated, but was not detected for the OH- and COOH-terminated surfaces. The absence of extra peaks in the retracting part of the FD curves (indicative of the presence of surface contaminants on the AFM tip and/or surface being interrogated) for gold AFM cantilevers interacting with CH_3_- and NH_2_-terminated SAMs confirm the effectiveness of the protocol employed for cleaning the AFM tips and SAM surfaces used in this study. The weak adhesion between the hydrophilic gold AFM tip and the hydrophobic CH_3_ SAM likely arises from interfacial tension^48^, whereas the considerably stronger interaction with NH_2_-SAM probably reflects electrostatic attraction between the opposing surfaces. In principle, the gold surface is electrostatically neutral, but in aqueous environments the adsorption of anions onto its surface lends it an overall negative charge^49–52^. In the present example, the adsorption of zwitterionic 4-(2-hydroxyethyl)-1-piperazineethanesulfonate buffer anions^53^ onto gold likely renders the latter surface negatively charged; which would explain (1) the greater force required to separate a bare gold AFM tip from the NH_2_ *cf.* CH_3_ SAM (i.e. 580 ± 107 *cf.* 143 ± 25 pN; Fig. 1b), and (2) the absence of any interaction between the bare AFM tip and the OH- and COOH-functionalized SAMs.

### Interaction between ‘gold-FNIII^8–14^’ AFM tips and SAMs

**Figure 3:**
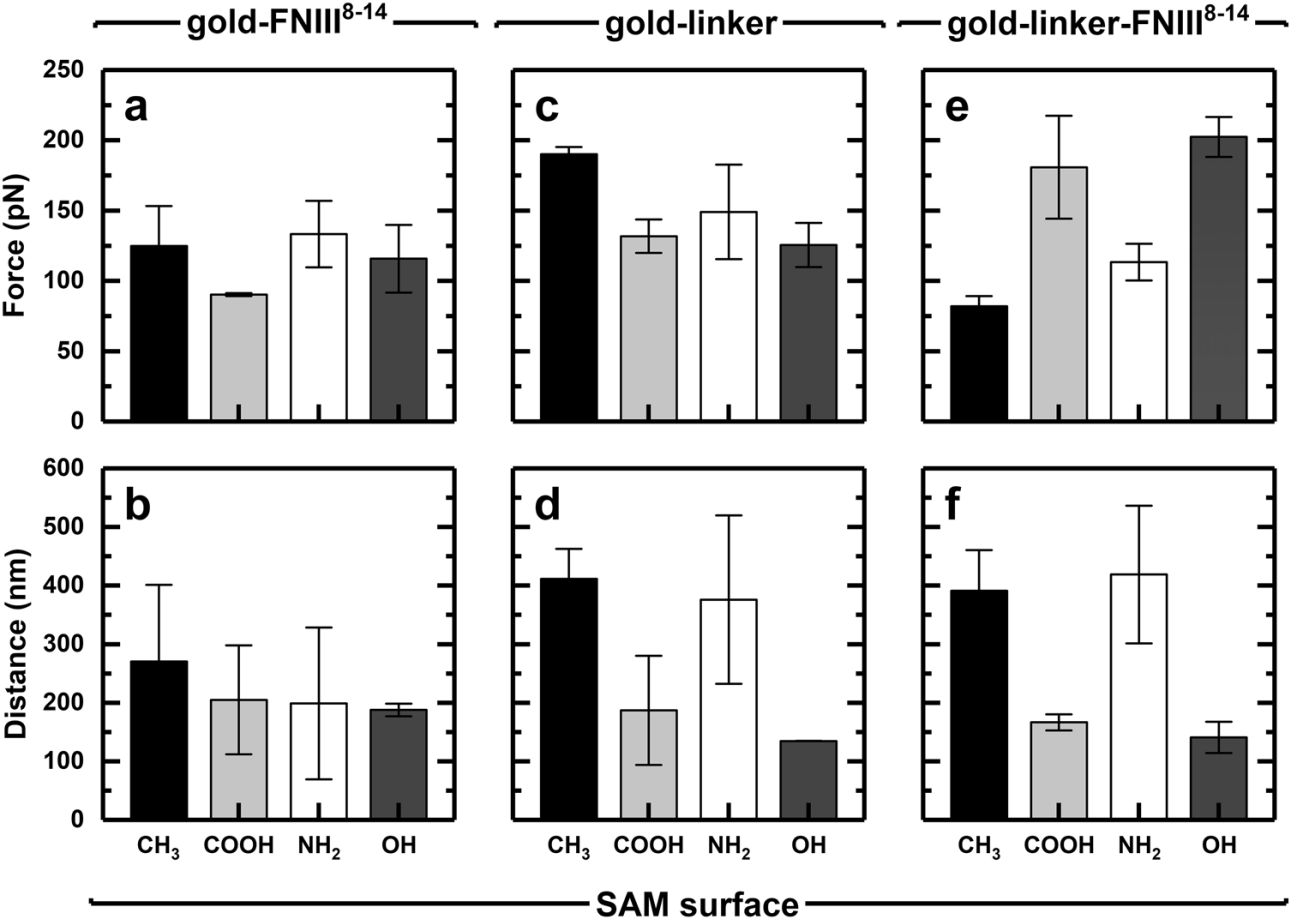
Plots of mean adhesion force (top) and desorption distance (bottom) for gold-FNIII^8–14^ (**a**, **b**), gold-linker (**c**, **d**) and gold-linker-FNIII^8–14^ (**e**, **f**) AFM tips against four different SAMs in 1 M HEPES buffer pH 7. The data represent the mean values ± standard error of n = 64 force curves. Statistical tests: P < 0.05 for (**a**, **d**–**f**), and P > 0.05 for (**b**, **c**).

In contrast to the single peaks observed in the FD retraction phase between bare gold and the CH_3_ and NH_2_ SAMs, multiple peaks were observed in the FD retraction curves between the physisorbed FNIII^8–14^ fragment and all four SAMs. For adsorbed proteins, the shape of retraction part of the FD curve is known to be dependent on: (1) the velocity of the AFM tip retracting from the surface; (2) the nature of the interactions between the protein and surface; and (3) protein conformation in the adsorbed state^54^. When multiple peaks arise (Fig. 2) the traces may exhibit one of three general forms, i.e. either: (1) a saw-tooth (loop-like) pattern, characteristic of unfolding of protein domains^55,56^; (2) an extended plateau (train-like) shape associated with desorption of proteins from charged or hydrophobic surfaces^57,58^, which has been attributed to denaturation of the adsorbed proteins^33^; or (3) some combination (mix) of (1) and (2). In physisorbed state, FNIII^8–14^ adopts different conformations at the gold AFM tip surface. A direct consequence of the conformational heterogeneity of FNIII^8–14^ in interactions of the gold-FNIII^8–14^ AFM tips with all four SAMs was no preference for a particular type of retraction force curve (Table 1). For three of the SAMs (CH_3_-, NH_2_-, COOH-terminated) the largest number of retraction FD curves (obtained with gold-FNIII^8–14^) were of the mixed type (41–64%). This aside, there were notable differences in the distribution of retraction curve shapes against the different SAM surfaces. For FD retraction curves measured against CH_3_ SAM, ~ 53% exhibited mixed events, ~ 41% showed train-like events and just 6.3% displayed loop-like curves. By contrast, none of the curves obtained with the OH SAM were train-like, 59% were loop-like, and 41% were of the mixed type. Given Meadows et al.’ correlation of train-like events with denaturation upon adsorption^33^, it follows that the extent of denaturation induced on FNIII^8–14^ by interaction with the SAM surfaces assumes the order CH_3_ > NH_2_ > COOH > OH. The desorption forces developed between gold-FNIII^8–14^ AFM tips and SAM surfaces are shown in Fig. 3a, and the corresponding desorption distances are presented in Fig. 3b. While the strength of adhesion between FNIII^8–14^ and the SAMs followed the order NH_2_ (135 pN) > CH_3_ (125 pN) > COOH (115 pN) > OH (~ 90 pN), ANOVA tests for the matching desorption distance data revealed no significant difference (P > 0.05) between the different SAMs (all ca. 200 nm).

**Table 1:** Distributions of ‘loop-like’, ‘train-like’ and ‘mixed’ conformations calculated from FD curves generated between differently functionalized AFM tips and SAM substrates.

Substrate	Gold-FNIII^8–14^ AFM tip (physisorbed)			Gold-linker AFM tip			Gold-linker-FNIII^8–14^ AFM tip (chemisorbed)		
	% loop	% train	% mix	% loop	% train	% mix	% loop	% train	% mix
CH_3_ SAM	6.3	40.6	53.1	0.0	0.0	100	0.0	14.3	85.7
COOH SAM	20.0	16.0	64.0	26.7	0.0	73.3	75.8	0.0	24.2
NH_2_ SAM	34.8	21.7	43.5	3.6	39.3	57.1	0.0	18.6	81.4
OH SAM	58.8	0.0	41.2	23.1	0.0	76.9	94.3	0.0	5.7

### Interaction between ‘gold-linker’ AFM tips and SAMs

Force spectroscopy experiments performed with AFM tips pre-functionalized with a 10:1 mixture of 11-mercapto-1-undecanol and *tris*NTA-EG_3_-C_16_-SH linker yielded FD curves featuring multiple pulling events (Table 1). By far the greatest number of interactions (57.1–100.0%) between the gold-linker AFM tips and SAMs were of the mixed type. Roughly 25% of the retraction curve shapes against the OH and COOH terminated surfaces were loop-like, whereas FD curves for the NH_2_ surface were the only ones to suggest a train-like conformation (40%). The magnitude of the desorption forces developed against all types of surfaces were of the order of 150 pN (Fig. 3c), but non-specific events likely arising from the presence of aggregates resulted in there being no significant differences (P > 0.05) between them in terms of force. By contrast, significant differences (P < 0.05) in desorption distances for the various surfaces were found (Fig. 3d), i.e. desorption distances for the CH_3_ and NH_2_ surfaces were roughly two and three fold greater than those for COOH and OH SAMs, respectively. Collectively, the above observations suggest that the pulling events detected in FD curves might arise from the presence of aggregates (derived from 11-mercapto-1-undecanol and/or *tris*NTA-EG_3_-C_16_-SH) with both hydrophobic and negatively charged regions sitting on top of the mixed ‘OH/linker’ SAM of the AFM tip post functionalization. The greater desorption distances observed with CH_3_ and NH_2_ terminated SAMs may reflect the presence of larger aggregates on these surfaces and/or greater unfolding of the entangled aggregates.

### Interaction between ‘gold-linker-FNIII^8–14^’ AFM tips with SAM surfaces

In contrast to findings with physisorbed FNIII^8–14^, most (i.e. > 82 to 100%) of the FD retraction curves for chemisorbed FNIII^8–14^ were loop-like or mixed varieties (Table 1), highlighting a specific interaction arising from successful functionalization. The probability of acquiring a force curve with meaningful feature, as defined in Fig. 2, was 57, 52, 69, and 55% for CH_3_, COOH, NH_2_, and OH-terminated SAMs, respectively. Predominantly loop-like curves were observed with COOH and OH SAMs (i.e. 76 and 94%, respectively) inferring domain unfolding. In contrast, CH_3_ and NH_2_ SAMs surfaces favored curves of mixed type, indicative of ‘denaturation and unfolding’. The presence of train-like curves, plus complete absence of loop-like ones, in interactions between chemisorbed FNIII^8–14^ and the CH_3_ and NH_2_ SAMs, infer that these two surfaces induce greater denaturation of the fibronectin fragment *cf.* the COOH and OH terminated varieties; the extent of denaturation induced assumes the order: NH_2_ > CH_3_ > COOH > OH.

**Figure 4:**
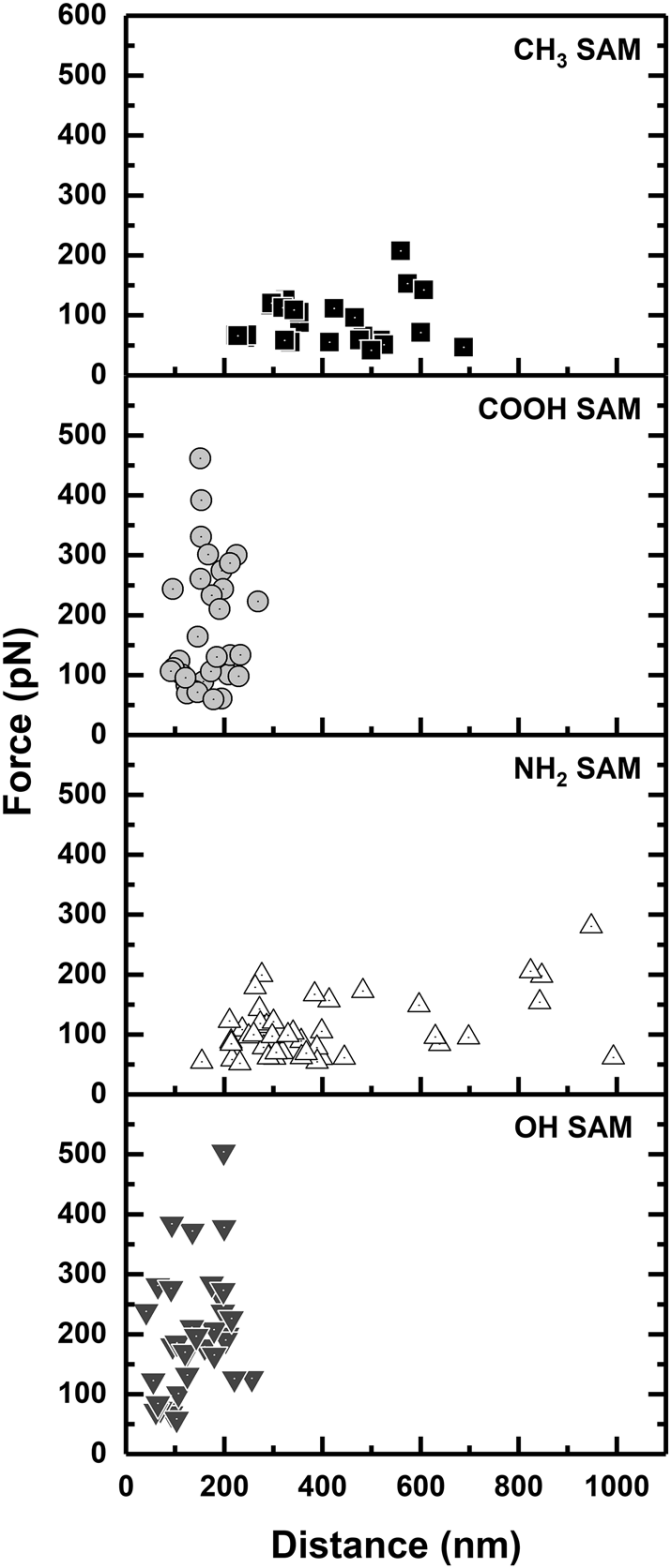
Force vs. distance maps for gold-linker-FNIII^8–14^ AFM tips against CH_3_, COOH, NH_2_ and OH terminated SAMs in 1 M HEPES buffer pH 7.5.

Of particular importance were striking differences between physisorbed and chemisorbed FNIII^8–14^. Whereas physisorbed FNIII^8–14^ showed a preference for CH_3_ and NH_2_ terminated surfaces (Fig. 3a), chemisorbed FNIII^8–14^ exhibited a contrary bias, i.e. substantially higher affinity for COOH and OH functionalized SAMs. For example, Fig. 3e shows that 2–3-fold greater desorption forces were required to release FNIII^8–14^ from COOH and OH *cf.* CH_3_ and NH_2_ terminated varieties, indicating that adsorption affinity for FNIII^8–14^ follows the order: OH ≥ COOH > NH_2_ > CH_3_. The exact opposite trend was noted for desorption distance, i.e. CH_3_ ≥ NH_2_ > COOH > OH (Fig. 3f). Maps of desorption force versus distance (Fig. 4) show loose clustering of the data obtained for each surface (from left to right as follows: OH, COOH, NH_2_, CH_3_), and serve to highlight the greater spread in desorption force *cf.* distance for OH and COOH surfaces, and the opposite for NH_2_ and CH_3_ SAMs, i.e. wider distribution in desorption distance *cf.* force. It is worth noting that previous study confirmed that the linker, tris-NTA-His6, remains intact over a range of adhesion forces up to 600 pN, which provides reassurance that the pulling events observed were not compromised^59^.

Given the differences in interaction types noted between the four SAMs, it follows that the conformations of FNIII^8–14^ adopted on these surfaces are likely different. The strength of interaction between a given protein and surface typically correlates with extent of unfolding/denaturation and ‘protein-surface’ contact area, i.e. the greater the interaction strength, the greater the unfolding, the larger the contact area^60,61^. The contact area will be largest when FNIII^8–14^ is orientated ‘side-on’ to the binding surface (long axis parallel to surface), and lowest when adsorbed in a ‘head on’ orientation (long axis perpendicular to the surface).

Under the experimental conditions applied, the surface of FNIII^8–14^ exhibits a net negative charge (ζ = − 14 ± 1 mV). FNIII^8–14^ binding to the positively charged surface of NH_2_ SAM is likely driven by large numbers of strong electrostatic attraction forces over a high contact area. A large footprint is also expected for FNIII^8–14^ on CH_3_ SAM the most hydrophobic surface (displaying a contact angle of 101 ± 1°) promoted by unfolding, exposure of and strong interaction with previously buried hydrophobic residues. Weaker forces steer the adsorption of FNIII^8–14^ on COOH and OH SAMs, i.e. van der Waals in the case of the OH SAM, and a combination of electrostatic and van der Waals interactions for the COOH SAM. Despite carrying a net negative charge positively charged regions within FNIII^8–14^ are attracted and eventually adsorbed on the COOH SAM surface. It is expected that OH and COOH surfaces have fewer contact points with the protein cf. CH_3_ and NH_2_ surfaces. We therefore envisage that a large fraction of FNIII^8–14^ molecules adsorb ‘side-on’ (i.e. with their long axis approximately parallel to the surface) on CH_3_ and NH_2_ SAMs, and ‘head-on’ (i.e. long axis approximately perpendicular) on the OH and COOH SAMs. The lower mean adhesion forces on CH_3_ and NH_2_ *cf.* OH and COOH SAMs (Fig. 3e) imply that these two surfaces induce less denaturation of FNIII^8–14^ (careful inspection of FD retraction curve data in Table 1 shows the reverse is true), and they also appear at odds with the above assumptions on the forces governing FNIII^8–14^ binding and its orientation in the adsorbed state. It should be stressed that numbers for the desorption force derive from the last peak in the retraction curve (Fig. 2). As FNIII^8–14^ molecules adhering to CH_3_ and NH_2_ surfaces are desorbed and unfolded over larger distances *cf.* COOH and OH SAMs, smaller numbers of them will remain on the surface before the final desorption force is determined, resulting in lower measured values.

### QCM studies

**Figure 5:**
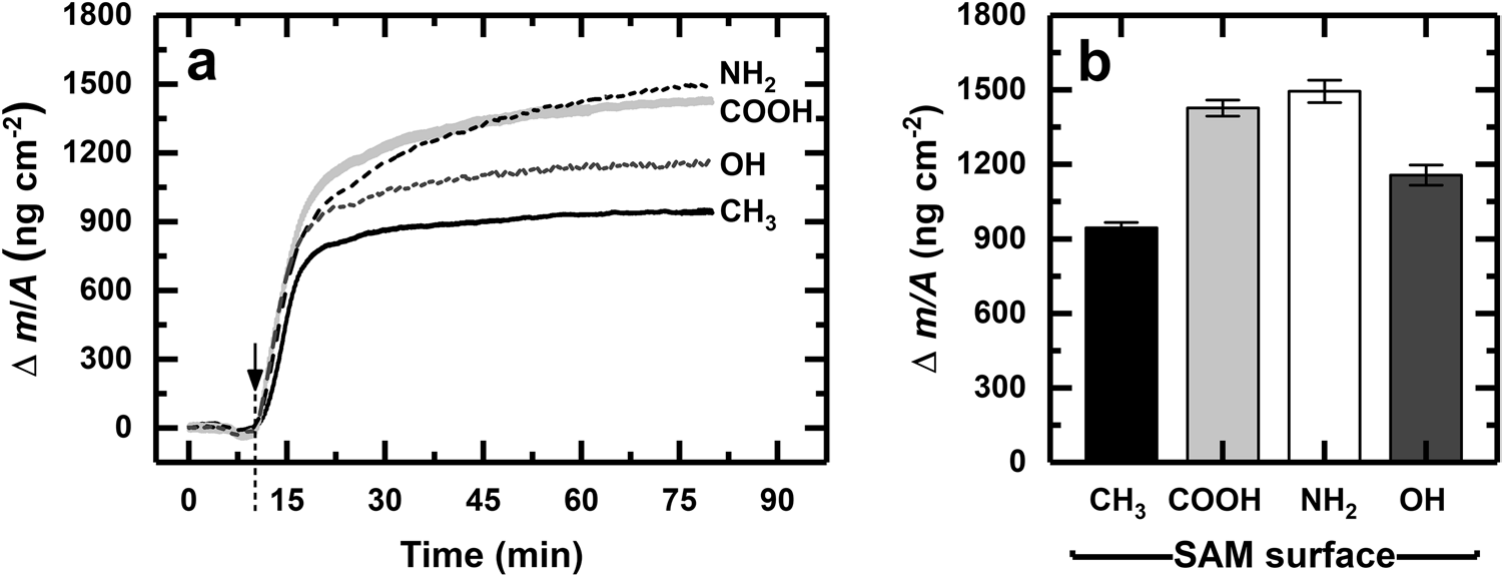
Plots of (**a**) change in surface concentration vs. time and (**b**) maximum surface concentration attained for the adsorption of fibronectin (25 µg/mL) on CH_3_–, COOH–, NH_2_– and OH-terminated SAMs in PBS buffer pH 7.4. The dip in the traces preceding the onset of binding (↓) reflects a momentary halt in pumping on switching from PBS to the protein solution. The data represent the mean values ± standard error of n = 7 measurements.

Figure 5a illustrates the time-dependent adsorption of full-length human FN onto gold-coated QCM crystals homogeneously functionalized with SAMs. At the relatively high FN concentration employed (25 µg mL^−1^), at least two kinetic steps can be discerned from the curves. Hydrated mass loads on the sensors increased rapidly over the first 5 min at broadly similar rates, i.e. ~ 130 ng cm^−2^ min^−1^ for the hydrophobic CH_3_ SAM and ~ 150 ng cm^−2^ min^−1^ for the three hydrophilic SAMs. Thereafter, adsorption rates diminished variably with increasing time, as surface occupancy increased and quasi-steady-state saturation was approached. At the very final stage (i.e. after 70 min of adsorption), surface concentrations (Fig. 5b) reached levels of 945 ± 22, 1158 ± 41, 1427 ± 32 and 1495 ± 45 ng hydrated mass per cm^2^ for the CH_3_, OH, COOH and NH_2_ SAMs respectively.

**Figure 6:**
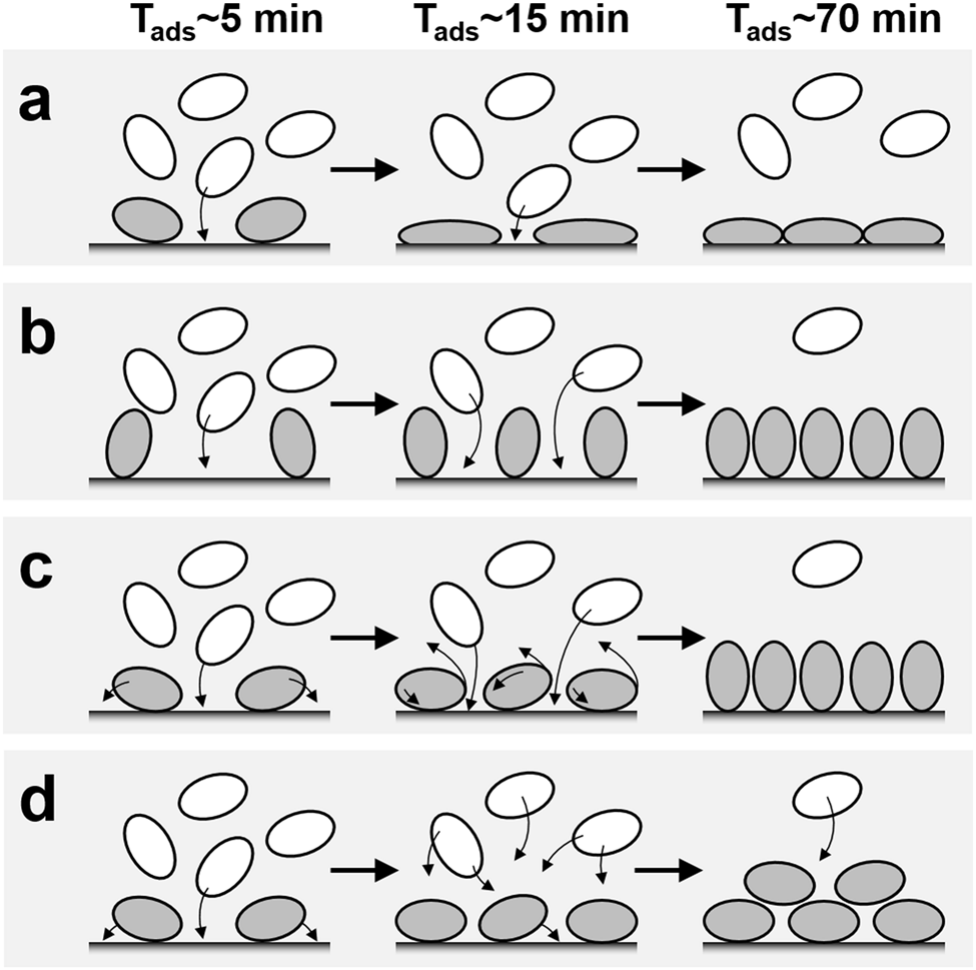
Schematic illustrations (**a**–**d**) showing possible changes in FN orientation and shape upon surface adsorption as a function of increasing time/surface coverage. The white and gray ovals represent free and bound FN respectively. (**a**) ‘Side-on’ binding with possible spreading forming a sparse monolayer; (**b**) ‘end-on’ monolayer binding; (**c**) initial ‘side-on’ binding switching to ‘end-on’ oriented monolayer; and (**d**) initial ‘side-on’ monolayer binding to multilayer.

FN is an extremely flexible molecule^62–64^ with a conformation strongly dependent on its local environment^65,66^, and ability to adsorb on very different surfaces and adopt different conformations at the interface^67–70^. Comparative studies of FN binding to hydrophilic and hydrophobic surfaces show that under normal physiological conditions, adsorption on hydrophobic, but not hydrophilic, substrates induces significant unfolding and footprint spreading^64,67–69,71,72^. The degree to which these events occur depends on: (1) the hydrophobicity of the surface, i.e. the more hydrophobic the greater the effects^72^; and (2) the bulk phase FN concentration and packing density (low surface coverage promotes strong unfolding and molecular expansion, whereas high packing density suppresses both effects)^64^. It follows that the lowest hydrated mass/area loading observed in this work for CH_3_ SAM (945 ng cm^−2^) likely reflects monolayer binding dominated by a space filling ‘side on’ orientation, with the additional possibility of an increase in FN’s molecular footprint (Fig. 6a). The significantly raised loadings on the OH (1157 ng cm^−2^) and especially COOH (1427 ng cm^−2^) SAMs *cf.* that on the methyl terminated SAM could be explained by greater numbers of FN molecules binding ‘end-on’ (Fig. 6b), in keeping with our findings in this work (Table 1, Fig. 4) of the single FNIII^8–14^ molecule’s observed preference for this orientation on these two surfaces.

Whilst binding to CH_3_ and OH SAMs involves short-range intermolecular forces, long-range electrostatics steer initial binding of FN to hydrophilic charged SAMs^73^. At first glance, the similar maximum loadings of FN on the negatively charged COOH and positively charged NH_2_ SAMs (1427 ± 32 and 1495 ± 45 ng cm^2^, respectively), may be difficult to reconcile with the molecule’s small net negative charge (ζ = − 5.7 mV) in PBS pH 7.4^35^. They are however, consistent with previous studies; indeed Lin et al.^74^ observed higher mass loadings on COOH *cf.*NH_2_ SAMs. Whereas FN binding to the amine-terminated surface is driven by electrostatic attraction, its adsorption on the carboxyl-terminated surface appears more complex, probably involving one or more positively charged residues, e.g. Lys 1469 located in a positive patch around the cell binding region^73^, augmented by shorter range holding forces^74^. Though similar surface concentrations were reached on NH_2_ and COOH SAMs the shape of the NH_2_ SAM binding profile implies a more complex adsorption behavior *cf.* the other surfaces. Here we envisage two possibilities. In both, FN binds initially in space-filling side-on manner commensurate with the single molecule’s preference. With increasing supply to the surface, adsorbed FN molecules either rearrange, tilting to a space-saving end-on configuration to maximize packing density (Fig. 6c), or alternatively multi-layer binding of FN (Fig. 6d).

## Conclusions

Knowledge on the adsorption behavior of FN molecules against a quartet of chemical defined surfaces was acquired by AFM-SMFS and QCM investigations. The following general conclusions can be drawn from the SMSF studies under physiological conditions: that extent of the unfolding and denaturation, strength of adsorption and binding orientation of FNIII^8–14^ correlated closely with one another. While the single molecule’s preference on more strongly adhering and denaturing positively charged NH_2_ and hydrophobic CH_3_ surfaces appears to be binding in a ‘side-on’ orientation, an ‘end-on’ configuration is favored on the comparatively gentle (i.e. less denaturing) more weakly interacting hydrophilic neutral OH and negatively charged COOH surfaces. The preferred binding orientation of the single tethered FNIII^8–14^ molecule was also inferred for the initial binding of free full-length FN on gold-coated QCM crystals modified with the same SAMs, i.e. ‘end-on’ on OH and COOH surfaces, and ‘side-on’ on CH_3_ and NH_2_ SAMs; in accord with Michael et al.’ findings^36^. In the context of cell binding, the orientation of surface adsorbed FN molecules is of key import, given that it determines the accessibility or otherwise of RGD motifs, which in turn influence cell surface interactions on exposure to the biological environment and during wound healing^3,4,6,7,9,11,75–80^. The approaches detailed herein on the interrogation of FN binding to chemically defined SAMs could be usefully employed as quantitative tools in much broader context including but not limited the design/development of surfaces that control cell adhesion and platelet activation, bacterial colonization, or limit fouling^78,81,82^.
